# The dual role of rivers in facilitating or hindering movements of the false heath fritillary butterfly

**DOI:** 10.1186/s40462-015-0031-z

**Published:** 2015-02-16

**Authors:** Henna Fabritius, Katja Rönkä, Otso Ovaskainen

**Affiliations:** Department of Biosciences, University of Helsinki, P.O. Box 65 (Viikinkaari 1), FI-00014 Helsinki, Finland; Current Address: Department of Biological and Environmental Science, University of Jyvaskyla, P.O. Box 35 (Survontie 9 C), FI-40014 Jyväskylä, Finland

**Keywords:** Habitat-specific models, Riparian corridors, False heath fritillary, *Melitaea diamina*

## Abstract

**Background:**

Species movement responses to landscape structures have been studied using a variety of methods, but movement research is still in need of simple methods that help predicting and comparing movements across structurally different landscapes. We demonstrate how habitat-specific movement models can be used to disentangle causes of differentiated movement patterns in structurally different landscapes and to predict movement patterns in altered and artificial landscapes. In our case study, we studied the role of riparian landscapes to the persistence of the endangered false heath fritillary butterfly (*Melitaea diamina*) in its newly discovered coastal distribution region in Finland. We compared the movement parameters of the riparian population to two reference populations by using capture-recapture data and habitat-specific diffusion modelling, and analysed the role of the river and riverbank buffer zones in facilitating or hindering false heath fritillary movement with movement simulations.

**Results:**

The riparian population of the false heath fritillary did not show major differences to reference populations in terms of movement parameters within breeding habitat, high-quality matrix and low-quality matrix. However, movement simulations showed that the habitat-specific movement parameters estimated for the false heath fritillary can lead into markedly different movement patterns in structurally different landscapes. An artificial riparian landscape mimicking those of the coastal distribution resulted into more directional, longitudinal movements both parallel and perpendicular to the river than a more mosaic-like landscape, but the existence of the river in the landscape reduced movements across the river.

**Conclusions:**

Our study demonstrates how habitat-specific movement models enable comparisons of movement patterns across structurally different real, altered and artificial landscapes. As such, they can be used to compare movement parameters across populations, to study the effects of management interventions to endangered species and to identify areas that have high sensitivity to individual movement. In our case study, the river is shown to perform a dual role for the movements of the riparian false heath fritillary population. Whereas the river acts as a moderate movement barrier for the false heath fritillary, the longitudinal configuration of riverbank habitats provides a means especially for the male false heath fritillaries to move across the landscape.

**Electronic supplementary material:**

The online version of this article (doi:10.1186/s40462-015-0031-z) contains supplementary material, which is available to authorized users.

## Background

Studies on species movement responses to landscape structures increase our understanding on how habitat fragmentation, human-generated landscape structures or management interventions affect individual movement [1], landscape connectivity or population viability. An example of a landscape structure in which movements have been studied by many researchers are riparian corridors, which provide habitats and migration pathways across human-modified landscapes for a large group of species and thus contribute to the conservation of terrestrial wildlife [2-4]. Riverbank buffer zones, which have historically been left astray because of susceptibility to flooding [3], are nowadays often protected by or managed according to environmental regulations [5,6]. Riverbanks are also influenced by high levels of soil moisture [7] and flood-based natural disturbance dynamics that can maintain a continuum of successional habitats [8-11]. Thus they may provide a variety of habitats ranging from riparian forest stripes [12] to wet open meadows [2].

Species occurrence and movements in riverbank habitats have been studied in several taxa, including moths [13], spiders [14], forest birds [12,15] and mammalian predators [16]. Among butterflies, many species inhabiting moist open habitats have been found to occur or move along riverbanks, like the Monarch butterflies [17], the Clouded Apollo [18] and the St. Francis satyr butterfly [19]. Study methods have included direct observations of movement [12,16,20,21], capture-recapture methods [17], spatiotemporal patterns in species sightings during migration periods [17], densities of species sightings at riverbanks versus other habitats [13,15,18] and population genetic studies [14,19]. Other studies have shown that the ability and willingness to cross rivers vary between species and taxa [22-25] and that the hostility of the river to the species has an effect on the likelihood of river crossings [26-28]. Relocation and mobbing call experiments have been used to study movements parallel and perpendicular to rivers in birds [29-32], demonstrating differences in species movement along rivers versus across them [33] and indicating the dual role of rivers in the formation of both movement barriers and functional movement corridors [34-37] for many terrestrial species.

Experimental data on species movement responses to tested landscape structures can be analysed by straightforward statistical analyses and can give reliable estimates on the effects of such structures on species movement. In contrast, making inferences on species’ movement in alternative landscapes is often not possible, since either the data lack the overall landscape context or the analysis model does not estimate the overall decision-making logic of the individuals studied. Movement researchers have expressed a need for a larger variety of methods that enable predicting species’ movement patterns in structurally different landscapes based on observed data [32,36] besides the existing potential methods and studies [36-39].

Habitat-specific movement models can be used to infer species’ movement parameters indirectly from capture-recapture data and habitat type maps of the study landscape [40-42]. In this study, we demonstrate how they can be used to disentangle causes of differentiated movements in structurally different landscapes and to predict movements in altered and artificial landscapes. Habitat-specific diffusion models have been successfully applied to non-territorial species with a presumably relatively simple movement logic, e.g. to some species of butterflies, the movement of which can be approximated by random walk at sufficiently large spatial scales [40-44]. Movement rate, measured by the diffusion parameter, is often expected to differ between at least three landscape types: breeding habitat (BH), high-quality matrix (HQM) and a more hostile low-quality matrix (LQM) [41,45,46]. Additionally, butterflies typically show edge-mediated behaviour [40,47-51], i.e. habitat selection at the edges between any two habitat types, which behaviour can be implemented in diffusion models by assuming that the probability of the butterfly being on preferred side of the edge is *k* times higher than the probability of it being in the other side of the edge, where the parameter *k* measures relative habitat preference [52].

**Figure 1:**
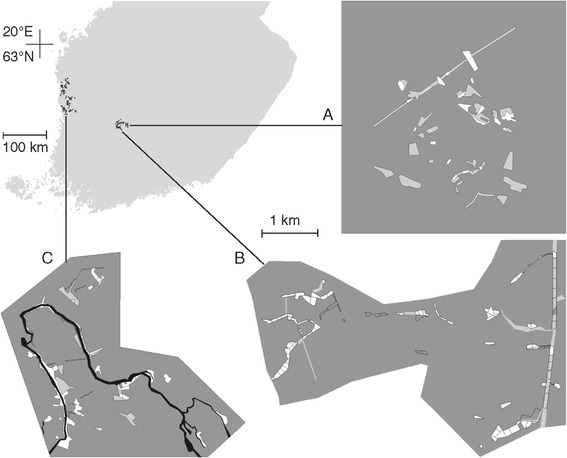
**False heath fritillary distribution in Finland and the landscape maps of the study areas.** The currently known distribution of the false heath fritillary in Finland is shown by the black dots in the main map. The landscape maps show the reference study areas REF1 (located in Siitama; **A**) and REF2 (Sorila; **B**) and the RIPARIAN study area (Merikarvia; **C**) on the same scale. We classified the landscapes into low-quality matrix (dark grey), high-quality matrix (light grey), breeding habitat (white) and the Merikarvia River (black). Lines in the low-and high-quality matrices depict boundaries of search areas.

The aim of our case study was to find out whether the structural configuration of riparian landscapes could be a key factor that explains the persistence of the endangered false heath fritillary butterfly *Melitaea diamina* (Lang, 1789) in its newly discovered coastal distribution region in Finland. The false heath fritillary is a moist meadow specialist that has, like many butterfly species, suffered from agricultural modernization and drainage of moist soils within the recent decades [53]. In Finland, sightings of the false heath fritillary have been made lately only in two distinctive regions of a narrow geographical range (Figure 1). The inland population has been subjected to various conservation measures, whereas the distribution along the West Coast of Finland was only properly revealed by a monitoring project in 2009–2012. It has remained unclear what has provided the means of persistence for the species at its newly discovered distribution.

We hypothesised that (H1) the coastal populations of the false heath fritillary are similar to the inland populations of the species in terms of habitat-specific movement parameters, but that (H2) the longitudinally structured riparian landscapes of the coastal distribution result into more directional, longitudinal movements than the mosaic-like landscapes of the inland populations. We assumed that the resulting connectivity across larger spatial scales could decrease metapopulation susceptibility to the effects of local climate variations, thus enforcing metapopulation persistence in the riparian landscapes [54,55]. To test the first part of our hypothesis (H1), we compared the habitat-specific movement parameters of the riparian coastal population to those of two inland reference populations (Figure 1A-C, Table 1) via posterior comparisons and by contrasting the riparian capture-recapture data against data simulated for the riparian landscape based on the reference movement models. Having verified the overall similarity of movement in the three populations, we tested the second part of our hypothesis (H2) by creating a generalised movement model for the false heath fritillary in Finland, using it to simulate movements in the riparian landscape and in altered and artificial landscapes and testing whether riparian landscapes would result into more directional, longitudinal movements than more mosaic-like landscapes.

**Table 1:** **Summary of the capture-recapture data sets**

**Population**	**Sampling period**	**Number of days sampled**	**Individuals captured**		**Individuals recaptured (total number of recaptures)**		**Estimated population size (standard error of the estimate) during sampling period**
			**Males**	**Females**	**Males**	**Females**	
REF1	16 Jun - 14 Jul 1995	25	555	273	298 (710)	151 (317)	1123 (29)
REF2	20 Jun - 6 Jul 2006	14	156	95	76 (118)	29 (47)	400 (26)
RIPARIAN	16 Jun – 11 Jul 2011	21	91	58	17 (23)	18 (24)	373 (64)

Summary of the capture-recapture data sets in the three study areas shown in Figure 1.

## Results and discussion

### Comparison of movement parameters across study areas

**Figure 2:**
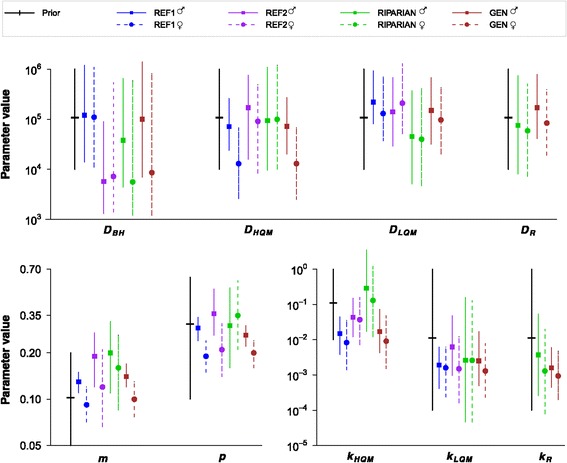
**Movement parameters of the false heath fritillary.** Marginal prior and posterior distributions of the model parameters among the three populations and those of the generalised movement model for Finland. Medians and 95% credibility intervals for males (females) are displayed with squares (circles) and continuous (dashed) lines. The parameter *k* (dimensionless) measures the habitat preference for a given habitat type relative to that in the breeding habitat, the diffusion parameter *D* (m^2^ day^−1^) measures the rate of movement, the parameter *m* (day^−1^) is the mortality rate, and the parameter *p* (dimensionless) is the capture probability. The subscripts BH, HQM, LQM and R stand for breeding habitat, high-quality matrix, low-quality matrix and the river.

Posterior comparisons of the model parameters (Table 2, Figure 2) did not show major differences in habitat-specific movement parameters between the riparian population and the two inland reference populations. In comparison to the reference populations, the riparian population shows a trend towards lower movement rate in the low-quality matrix (*D*_*LQM*_), higher preference for the high-quality matrix (*k*_*HQM*_), higher female mortality (*m*) and higher capture probability of females (*p*). However, the statistical support for these differences remained weak (Table 3), which is partly due to the large amount of posterior uncertainty in the riparian movement model (Figure 2). This was in turn caused by low population density during the year of the capture-recapture experiment in the riparian population, resulting in a low number of individuals and recaptures relative to the number of days sampled (Table 1). Thus, direct comparisons of movement parameters among the three populations were consistent, without any strong evidence for population specific movement parameters.

**Table 2:** **Parameters of the habitat-specific diffusion model**

**Parameter**	**Unit**	**Description**
*k*		Relative habitat preference, i.e. the relative probability of the butterfly being located in the habitat type in question in comparison to the probability of it being located in its breeding habitat (BH; *k*_*BH*_ = 1 by definition).
*D*	m^2^ d^−1^	The diffusion coefficient, i.e. the rate of movement within a given habitat type.
*m*	d^−1^	Mortality, measured as the probability of the butterfly dying during one day.
*p*		Capture probability, i.e. the probability of the butterfly being captured if it is located at the searched site.

**Table 3:** **Posterior comparison across populations and between sexes**

**Posterior comparison**		***k***_***HQM***_	***k***_***LQM***_	***k***_***R***_	***D***_***BH***_	***D***_***HQM***_	***D***_***LQM***_	***D***_***R***_	***m***	***p***
Differences across populations										
males ♂	P(RIPARIAN^♂^ > REF1^♂^)	0.973**	0.570	-	0.253	0.566	0.100*	-	0.906**	0.532
	P(RIPARIAN^♂^ > REF2^♂^)	0.912**	0.532	-	0.920**	0.547	0.416	-	0.673	0.471
females ♀	P(RIPARIAN^♀^ > REF1^♀^)	0.974**	0.600	-	0.092**	0.926**	0.201*	-	0.950**	0.982**
	P(RIPARIAN^♀^ > REF2^♀^)	0.800*	0.583	-	0.431	0.552	0.116*	-	0.731*	0.941**
Differences among sexes										
P(GEN^♂^ > GEN^♀^)		0.722	0.721	0.704	0.846*	0.946**	0.656	0.739	0.991**	0.984**

Bayesian posterior probabilities of habitat-specific movement model comparisons across populations and between sexes. RIPARIAN refers to the riparian population, REF1 and REF2 to the two reference populations and GEN to the generalised movement model (see “Methods”). Posterior probabilities higher than 0.75 (or lower than 0.25) are marked with (*) and those higher than 0.90 (or lower than 0.10) are marked by (**).

**Figure 3:**
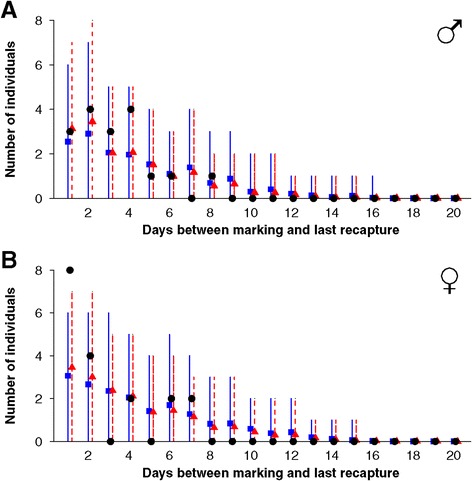
**Days between marking and last recapture in the riparian population in comparison to the predictions by the reference models.** Black circles depict real riparian data; blue squares (red triangles) with continuous (dashed) error bars show the means and 95% credibility intervals of posterior predictive data simulated for the riparian landscape based on the parameters estimates of the reference population REF1 (REF2). The data are shown separately for males **(panel A)** and females **(panel B)**.

**Figure 4:**
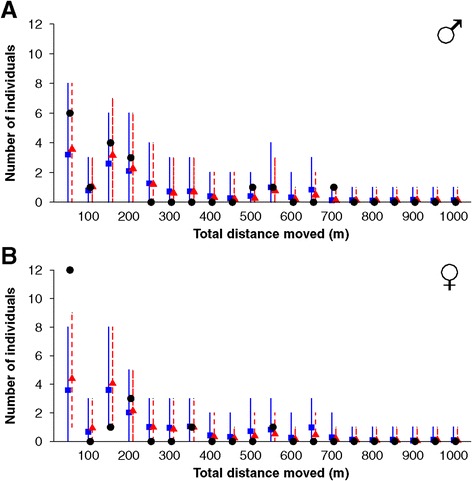
**Total distance moved in the riparian population in comparison to the predictions by the reference models.** Black circles depict real riparian data; blue squares (red triangles) with continuous (dashed) error bars show the means and 95% credibility intervals of posterior predictive data simulated for the riparian landscape based on the parameters estimates of the reference population REF1 (REF2). The data are shown separately for males **(panel A)** and females **(panel B)**.

False heath fritillary movement parameters proved similar across populations also when the riparian capture-recapture data was contrasted against data simulated for the riparian landscape based on the reference movement models (Figures 3 and 4, Table 4). Among the test statistics we used, the distribution of times between marking and last recapture (Figure 3) acts as a proxy for life-span, whereas the observed total movement distance (Figure 4) acts as a proxy for overall movement activity. The exception for the good fit between the riparian data and the predictions of the models parameterised by the reference populations is the number of females that were observed only during one day or which moved less than 50 m in total between marking and last recapture, which number was underestimated by the model predictions (Figures 3 and 4, Table 4).

**Table 4:** **Comparison of the riparian capture-recapture data against predictive posterior data simulated based on the reference movement models**

		**Riparian data**	**Prediction (REF1)**	**Prediction (REF2)**
Recaptured individuals	All	35	34.6 (24–47)	34.1 (22–47)
	Males	17	16.4 (8–26)	16.5 (7–28)
	Females	18	18.1 (11–25)	17.6 (10–25)
Number of recaptures	All	47	42.9 (28–59)	42.8 (26–63)
	Males	23	19.1 (9–31)	19.5 (7–36)
	Females	24	23.8 (13–35)	23.2 (12–36)
Recaptures in the location of previous capture	All	28	21.2 (12–31)	19.9 (11–30)
	Males	12	9.8 (4–17)	9.4 (3–16)
	Females	16	11.3 (6–17)	10.7 (5–17)
Recaptures in a different location	All	7	13.5 (6–21)	13.9 (6–24)
	Males	5	6.5 (2–12)	7.1 (2–15)
	Females	2	6.8 (2–12)	6.9 (2–13)
River crossings	All	4	7.5 (2–13)	8.4 (2–17)
	Males	3	3.6 (0–8)	4.3 (0–11)
	Females	1	3.9 (0–8)	4.2 (0–9)

The table shows the number of individuals in the real riparian data that fall into each category and the posterior means (and 95% credibility intervals) of posterior predictive data simulated based on the parameters estimated for the reference populations The river has been assumed as low-quality matrix in the REF1 and REF2 predictions.

The above results suggest that false heath fritillary populations are not differentiated in a major way in terms of their movement behaviour across the two distribution regions. The relatively small observed differences across distribution regions are more likely to be caused by variation in the habitat types (which were classified here in three very broad categories), or differences in weather conditions during the capture-recapture experiments, rather than e.g. genetic or other biologically important differences.

### Movement parameters in the two sexes and across habitat types

A generalised movement model for the false heath fritillary in Finland, created by pooling the riparian and REF1 data sets, provided relatively narrow posterior distributions especially for mortality, capture probability and habitat type preference (Figure 2). It differed from the model parameters of REF1 (Figure 2) mostly for the movement rate in the breeding habitat (*D*_*BH*_), for which the original REF1 data did not contain information due to its capture-recapture study design [40]. According to this model, males moved faster than females in the breeding habitat (*D*_*BH*_) and high-quality matrix (*D*_*HQM*_), and had higher mortality rates (*m*) and capture probabilities (*p*; Figure 2 and Table 3), suggesting a median expected life-time (1/*m*) of ten days for females and seven days for males. Both sexes showed higher preference for and had lower movement rates in the breeding habitat in comparison to the high-quality matrix  and showed higher preference for the high-quality matrix in comparison to the low-quality matrix . Females also followed the expectation [21] that the rate of movement is faster in the low-quality matrix than in the breeding habitat . These differences are in line with the previous view of the false heath fritillary as a rather sedentary butterfly, with males patrolling for females for mating and thus being slightly more mobile than females [56-60].

In the generalised movement model and in the riparian movement model, estimated habitat preference (relative to breeding habitat) for the river (*k*_*R*_) were markedly lower in comparison to the preference of high-quality matrix (*k*_*HQM*_); there was only a small overlap between the posteriors in both models and a nearly hundred-fold difference between the medians in the riparian model (Figure 2). Estimated habitat preference for the river (*k*_*R*_) also had slightly lower medians and narrower posterior distributions in comparison to those of the low-quality matrix (*k*_*LQM*_; Figure 2), but these differences did not gain much statistical support in posterior comparisons . In line with this, the number of river crossings in the riparian data was below the medians predicted by the reference movement models, in which movements across the river were predicted as if the river was part of the low-quality matrix (Table 4), but nevertheless within the 95% posterior credibility intervals. The results thus suggest that the river fits well within the low-quality matrix habitat type in terms of movement rates (*D*) and boundary responses (*k*).

**Figure 5:**
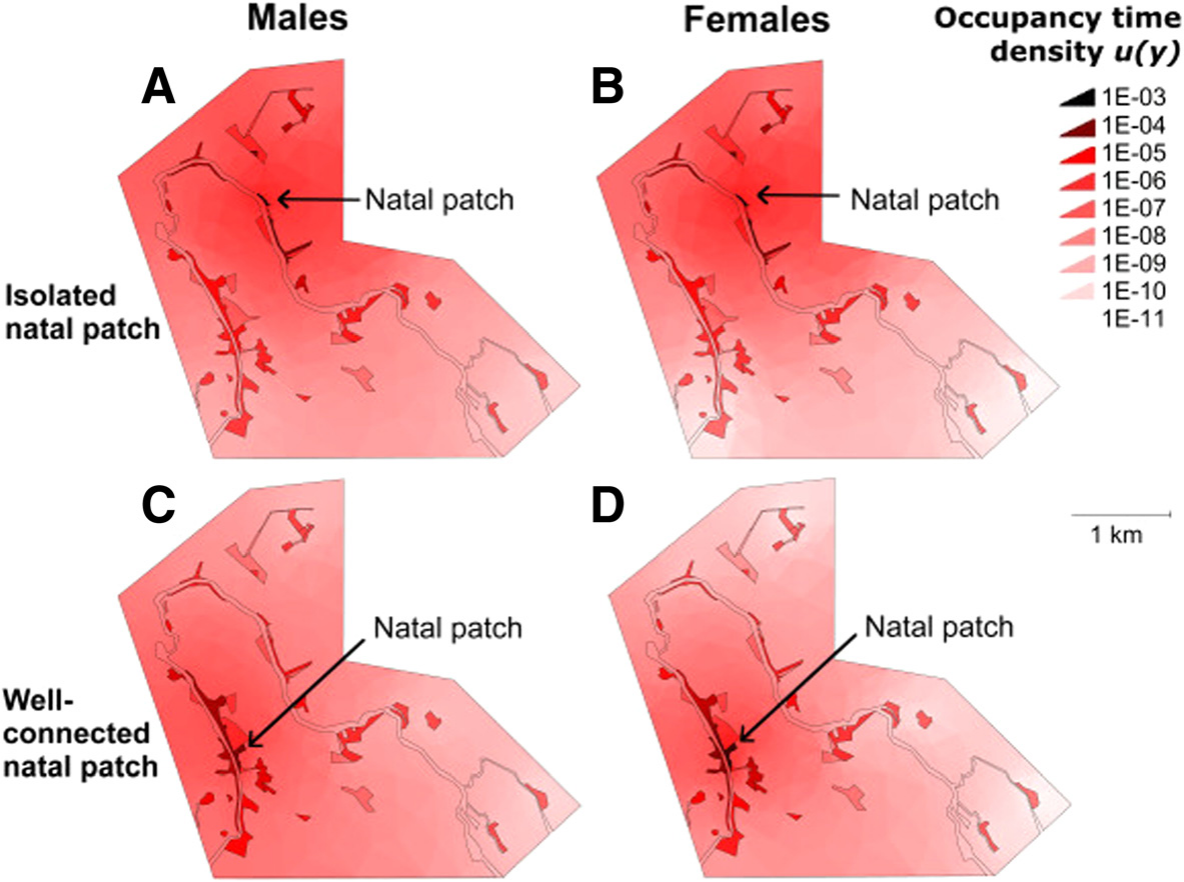
**Time spent by simulated false heath fritillaries at different locations of the study area.** The graph shows the posterior median estimate for occupancy time density (days m^−2^) that simulated male (left hand panels) and female (right hand panels) false heath fritillaries spend in any location of the landscape, if initially in the location pointed with an arrow. The results are displayed for an isolated habitat patch **(panels A-B)** and a well-connected habitat patch **(panels C-D)**.

A movement simulation based on the posterior medians of the generalised movement model (Figure 5) illustrates the sedentary nature of the false heath fritillary butterflies, individuals being likely to spend most of their time along a network of connected habitat patches (Figure 5, dark colours). Individuals starting flight at a well-connected patch (panels C-D) would be more likely to spend time in the habitat patch network than individuals starting movement at a more peripheral patch (panels A-B), who would end up spending some of their time in an area of the low-quality matrix that did not contain habitat patches (upper right). Female movement is somewhat more confined to the habitat patches than that of the males. These patterns highlight the importance of the well-connected habitat patch network provided by the riverbank buffer zones in the riparian landscape.

### False heath fritillary movement patterns in structurally different landscapes

**Figure 6:**
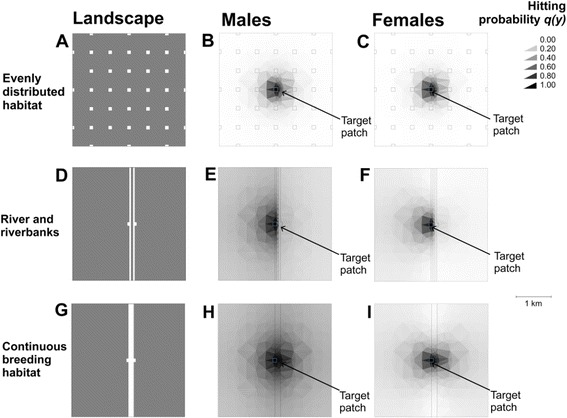
**The effect of the river and riverbank habitats to the movements of the false heath fritillary in a hypothetical landscape.** The graph shows probability of simulated male (middle column of panels) and female (right hand column of panels) false heath fritillaries to reach a target patch (light blue edges; pointed with an arrow, located on the Western side of the river) within its lifetime, as a function of its place of birth. The results are shown for a landscape with a 50-meter wide low-quality matrix stripe (the river) and symmetric riverbank habitats **(panels D-F)**, a similar landscape in which the river has been replaced with breeding habitat **(panels G-I)** and for a landscape where the same amount of habitat (in squares) and low-quality matrix as in the artificial riparian landscape has been distributed evenly across the landscape **(panels A-C)**. Parameter values set to the posterior median values of the generalised movement model. We classified the landscapes **(panels A, D, G)** into low-quality matrix (dark grey) and breeding habitat (white).

**Figure 7:**
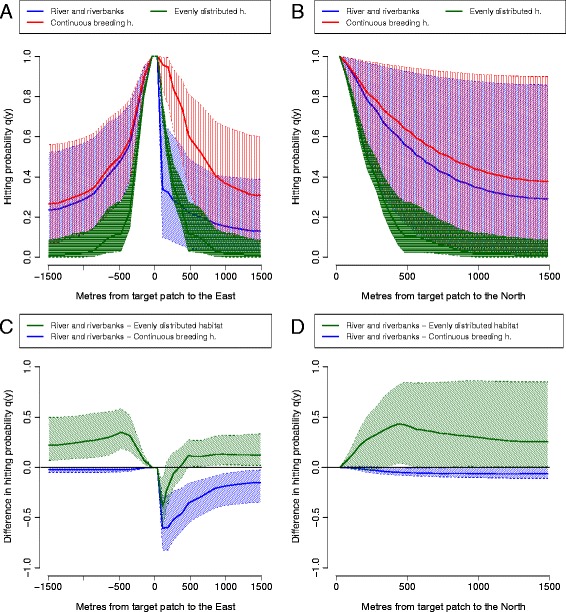
**The effect of parameter uncertainty on movement predictions for male false heath fritillaries.** The graph shows cross-sections of the hitting probabilities of simulated male false heath fritillaries in the artificial landscapes of Figure 6 parallel and perpendicular to the river **(panels A-B)** and the differences of the hitting probabilities between movements in artificial landscapes **(panels C-D)**. The medians and 95% credibility intervals are based on 100 random samples from the posterior distribution of the generalized movement model.

**Figure 8:**
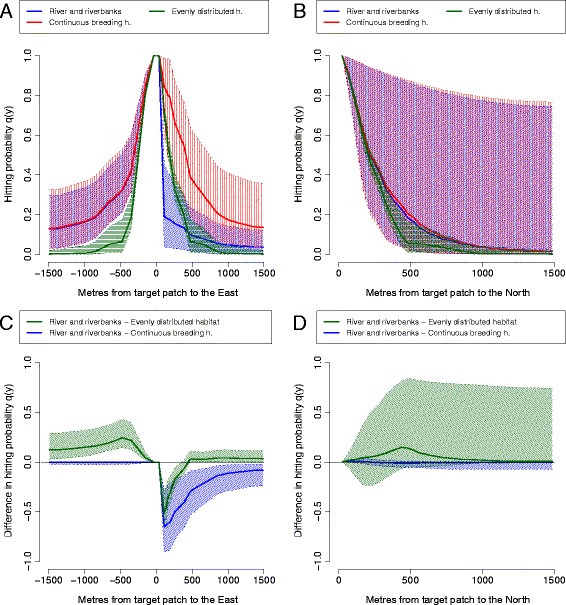
**The effect of parameter uncertainty on movement predictions for female false heath fritillaries.** The graph shows cross-sections of the hitting probabilities of simulated female false heath fritillaries in the artificial landscapes of Figure 6 parallel and perpendicular to the river **(panels A-B)** and the differences of the hitting probabilities between movements in artificial landscapes **(panels C-D)**. The medians and 95% credibility intervals are based on 100 random samples from the posterior distribution of the generalized movement model.

Movement simulations carried out using three artificial landscapes (Figure 6) demonstrate how different landscape elements interplay to inhibit or facilitate false heath fritillary movements in a riparian landscape. First, we compare false heath fritillary movement patterns between a riparian landscape consisting of a low-quality matrix river and symmetric riverbank habitats (Figure 6, panels D-F), and a control landscape where an equal amount of habitat and low-quality matrix is distributed evenly across the landscape (panels A-C). For male false heath fritillaries, the probabilities of hitting a target patch both from the North (parallel to the river) and from the East and West (perpendicular to the river) are higher in the riparian landscape than in a control landscape (Figure 7, panels C-D). Long directional movements perpendicular to the river occur due to the absence of habitat patches in the surrounding landscape, causing the males to fly fast across the low-quality matrix (*D*_*LQM*_) while searching for habitat patches. If habitat patches are distributed evenly across the landscape, males are likely to stop at habitat patches and may change direction when leaving a patch. Long directional movements parallel to the river are co-products of relatively high movement rates in, and a high preference of, the breeding habitat (*D*_*BH*_ and *k*_*BH*_). For female false heath fritillaries, differences in movement patterns are otherwise similar to those of males, except that probabilities of hitting a target patch from the North (parallel to the river) are not higher in the riparian landscape than in the control landscape due to lower estimated movement rates in the breeding habitat (*D*_*BH*_, Figure 8). Hitting probabilities diagonal to the river are likely to be higher than those parallel to the river for females (Figure 6, panel F).

We next compare false heath fritillary movement patterns between the riparian landscape (Figure 6, panels D-F) and a similar landscape where the river has been replaced with breeding habitat (panels G-I). The probabilities of hitting a target patch from the East (perpendicular to the river) are lower for both male and female false heath fritillaries in the riparian landscape (Figures 7 and 8, panel C) due to the low preference of entering the low-quality matrix environment (*k*_*LQM*_). For male false heath fritillaries, the probabilities of hitting the target patch from the North and from the West are also lower (Figure 7, panel D), probably because of faster movement in (*D*_*LQM*_) and lower preference of (*k*_*LQM*_) the river environment; individuals that enter the river environment are either likely to fly fast past the target patch or are not likely to cross the river for another time.

**Figure 9:**
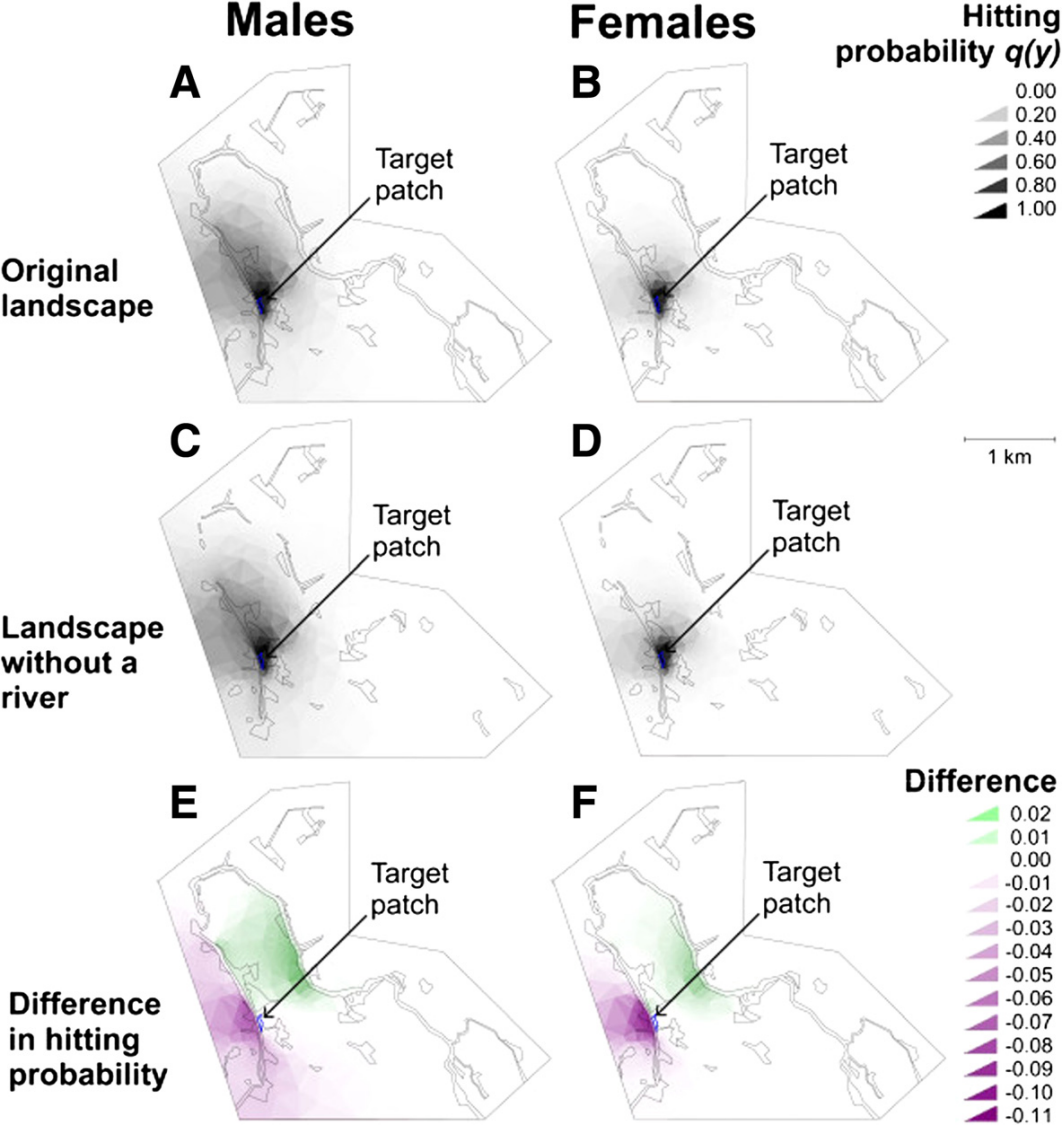
**The effect of the river to the movements of the false heath fritillary in the study landscape.** The graph shows probability of simulated male (left hand panels) and female (right hand panels) false heath fritillaries to reach a target patch (blue; pointed with an arrow, located on the Eastern side of the river) within its lifetime, as a function of its place of birth. The results are shown for the riparian landscape in which the river has been modelled as an independent habitat type **(panels A-B)** and another version of this landscape, in which the river has been modelled as part of the low-quality matrix **(panels C-D)**. The difference of the probabilities (results of panels A-B minus those of C-D) has been plotted separately **(panels E-F)**: from the green (purple) areas, the simulated false heath fritillaries are more (less) likely to reach the target patch if the river was modelled as an independent habitat type. Parameter values set to the posterior median values of the generalised movement model.

Movement simulation carried out in the riparian landscape of the riparian study area (Figure 9) visualises regions where nuanced changes in the species’ movement parameter estimates would have largest effects to estimated hitting probabilities. While differences are small between a landscape where the river was considered a distinct habitat type with its own (median) movement rates and boundary responses (panels A-B) and a landscape where the river is modelled as part of low-quality matrix (panels C-D), a more detailed comparison (panels E-F) demonstrates that they are likely to be strongest for individuals that start movement on the opposite side of the river from the target patch (decreasing their probability of hitting the target patch) and for individuals starting from the same side as the target patch but close to the opposite riverbank (increasing their possibility to hit the target patch). This demonstrates the role of the riverbends of the riparian landscapes in increasing and decreasing connectivity between habitat patches.

The above simulations demonstrate that a longitudinally structured riparian landscape performs a dual role for the movements of the riparian false heath fritillary population. First, as hypothesized, longitudinally structured riparian landscapes result into more directional, longitudinal movements than the mosaic-like landscapes, thus generating habitat connectivity for the riparian false heath fritillaries. However, the river itself acts as a structural movement barrier for the false heath fritillaries, reducing movements across the river. Such dual effect has implications for conservation planning: low probabilities of river crossings decrease connectivity between the two sides of the river but riverbank habitats can increase connectivity within each of the sides [23].

### Habitat-specific movement models as disentanglers of movement patterns from landscape structure

Our analyses show that habitat-specific movement parameters can lead into markedly different movement patterns in structurally different landscapes for some species. This is likely to be the case especially for habitat specialists, like the false heath fritillary, that spend most of their time in their breeding habitat. Habitat-specific movement models make it possible to predict the movements of such species in structurally different landscapes, and thus can be used to study the effects of management interventions, e.g. the placement of conservation sites, to the movements of endangered species and to identify areas in the landscape that have high sensitivity to their movement. The possibility to use also artificial landscapes for studying movements enable studying movements in structurally simple, symmetrical landscapes, which clarify the often complex effects of multiple landscape elements to movements, and series of slightly altered landscapes, which enables quantifying the effects landscape structure to individual movements.

Habitat-specific movement models can be parameterised using spatially explicit capture-recapture data, which kind of data are difficult to analyse with many other methods because it tells about movement behaviour only indirectly. Such data are often the only possibility for movement analysis for threatened species, for which relocation experiments may not be possible due to legislative restrictions. The indirect nature of capture-recapture data is an obvious drawback, both in terms of limited informativeness of the data as well as the need for more sophisticated analysis methods. For instance, cultivated fields and closed forests may be quite different environments for butterfly movement, but we have pooled them in the broader category of low-quality matrix based on prior analysis results [61], as the capture-recapture data lack resolution to disentangle if movements would differ among these habitat types. Similarly, it might be the case that the river environment differs from closed forests and cultivated fields in terms of butterfly movement, even though we could not prove such a difference in this study. The existence of such a difference would be most efficiently studied by relocation experiments, where butterflies would be followed to directly study their behaviour at habitat type edges, which unfortunately were not possible for our study species.

## Conclusions

Our study demonstrates how habitat-specific movement models and already a simple representation of landscape structure enable comparisons of movement patterns across structurally different real, altered and artificial landscapes. As such, they can be used to compare movement parameters across populations, to study the effects of management interventions and to identify areas that have high sensitivity to individual movement.

In our case study, the river is shown to perform a dual role for the movements of the riparian false heath fritillary population. As habitat specialists [62], the false heath fritillaries have a high tendency to stay at habitat patches and thus their movement is very much dictated by the spatial configuration of their habitat patch network. Whereas rivers act as moderate movement barriers for the false heath fritillary, the longitudinal configurations of riverbank meadows provide a means especially for the male false heath fritillaries to move across the landscape.

## Methods

### The study sites, capture-recapture data and habitat classifications

The reference populations REF1 (located in Siitama; 61.6°N, 24.2°E) and REF2 (Sorila; 61.55°N, 23.9°E) belong to the same metapopulation system [56-58] and are located 15 km from each other near the city of Tampere. The REF1 study site contains a dense cluster of 14 habitat patches and other meadows classified as high-quality matrix within a landscape of forests and cultivated fields (Figure 1A). The REF2 study site is characterized by more sparsely located stripes of habitat patches and high-quality matrix, including open areas such as a powerline right-of-way at the Eastern edge (Figure 1B). The riparian study site at Merikarvia (61.86°N, 21.56°E) is characterized by the ~30-50 m wide Merikarvia River that twists across the landscape (Figure 1C), with habitat patches and high-quality matrix along riverbanks and some powerlines. At all study sites, monthly mean temperatures vary from −8°C to 16.5°C, the mean annual rainfall is approximately 650 mm and a permanent snow cover lasts for 3–5 months [63].

Habitat-specific movement analyses were based on habitat classification maps (Figure 1) that categorized the landscape into breeding habitat (open or semi-open meadows with the host plant *Valeriana sambucifolia*), high-quality matrix (open areas with nectar plants), low-quality matrix (e.g. closed forests and cultivated fields) and the river. Butterfly searching took place within all three terrestrial landscape classes based on a discrete set of search sites, so that the search effort could be described as a table of sites searched each day.

The capture-recapture data for REF1 has been described by Wahlberg [59] and Wahlberg *et al.* [56,60] and the original habitat classifications by Ovaskainen [40; model B]. In REF2, capture-recapture data was originally collected and analysed by Ovaskainen and Cabeza for a non-refereed conservation study [61]. In REF2, 73, 12 and 27 polygons within the breeding habitat, the high-quality matrix and the low-quality matrix respectively with an average size of 0.57 ha were used as search sites. At this time point, the open areas (O) and forests (F) of REF1 were reclassified into areas of high-quality matrix and low-quality matrix (Figure 1). In the riparian population, we collected data using the EarthCape software [64]. We split the previously delineated 21 large habitat patches into search polygons, each with a diameter of 62 m in minimum, and placed 20 longitudinal search areas of width 15 m into the remaining landscape for the capture-recapture study. Six of these search areas were categorised as high-quality matrix and 12 as low-quality matrix during habitat classification. We estimated population sizes during the sampling periods using the open population model of Rcapture [65]. To account for births and deaths occurring during the sampling periods, and to find a model with smallest residuals, we grouped the capture-recapture data into two-day (REF2, RIPARIAN) or four-day (REF1) primary periods and, as instructed [65], removed outliers with standardized residuals greater than four before population size estimation. The details of the capture-recapture studies and the resulting data sets [66] are summarised in Table 1.

### Estimation of movement parameters

We applied habitat-specific diffusion modelling with Bayesian inference [40,41] to estimate posterior distributions of the model parameters separately for males and females. We triangulated the habitat classification and capture-recapture site maps with the Mapper software [42] and analysed the triangulated maps, the search effort matrix and the observation matrix with the Disperse software [42]. Disperse computes the likelihood of the data using the finite element method, and estimates posterior distributions via adaptive MCMC methods.

The biological parameters estimated were the habitat-specific diffusion coefficients *D* measuring the rates of individual movement, the habitat preference *k* for each habitat type relative to that in the breeding habitat (*k*_*BH*_ = 1 by definition), and the mortality rate *m*. The observation model involves the parameter capture probability *p*, i.e. the probability of capturing an individual from the search site conditional on the individual being present. We used lognormal priors for *k*, *D* and *m* and a logit-normal prior for *p*. The prior medians were derived from a long-term study of a closely related species *Melitaea cinxia* [67], and we assumed wide credibility intervals to account for interspecies differences. We assumed the same priors for the river as for the low-quality matrix. To create the generalised movement model (GEN) for the false heath fritillary across distribution regions, we estimated the movement parameters for the riparian data using the posteriors of the most data-rich REF1 population (Figure 2, (Additional file 1)) as priors, which corresponds to the estimation of the model parameters from the combined data sets.

### Comparison and cross-validation of parameter estimates

To assess the similarity of posterior distributions among the three populations and between the two sexes we calculated the posterior probability of difference by drawing 10000 pairs of random samples from each pair of the posterior distributions to be compared, as in Ovaskainen *et al.* [41]. Posterior comparisons between the two sexes were based on the generalised movement model.

To test whether the movement models of the reference populations would correctly predict false heath fritillary movements in the riparian landscape, we generated posterior predictive data by drawing 1000 samples from the reference model posterior distribution and simulated movements for each sample, assuming for the river (which was absent from the reference populations) the parameters of the low-quality matrix. We set the simulations to start at the locations of real butterfly marking events at the riparian landscape, generated a movement track for each individual, and generated capture-recapture data assuming the same spatiotemporal search effort as in the field study. We adjusted the capture probability *p* for both males and females so that the proportions of recaptured individuals matched with the real proportions in the riparian data. We compared simulated data against the real data with respect to the following parameters: the number of river crossings, the distribution of total distance moved by recaptured individuals, the distribution of times from marking until last recapture, and the ratio of migrated individuals. To retrieve parameter values for all individuals, we generated 1000 sums of random pairs of male and female results.

### Movement simulations

To analyse the roles of the high-quality matrix and the river for false heath fritillary movements, we created movement simulations for the riparian landscape based on the generalised movement model by using both the original, altered and artificial landscape maps. Simulated individuals either started flight from a pre-defined natal patch, after which we calculated the occupancy time density *u(y)*, the time that the individual was expected to spend at any location *y* of the study area during its lifetime [68]. Alternatively, we calculated the hitting probability *q(y)*, the probability that the butterfly starting its flight from any location *y* would reach a target patch during its lifetime [68]. We visualised the effect of the landscape alterations by plotting the difference of *q(y)* between the original and the altered landscape at each map location *y*. We created credibility intervals for the movement statistics by generating them for 100 parameter combinations sampled from the posterior distribution.

## Availability of supporting data

The data sets supporting the results of this article are available in the Dryad repository, doi:10.5061/dryad.j54vv, [http://doi.org/10.5061/dryad.j54vv].

## Additional file

Additional file 1:
**Prior and posterior distributions of the false heath fritillary.** An Excel file. The medians (and 95% credibility intervals) of the posterior distributions for false heath fritillary males and females across the three study areas. RIPARIAN refers to the riparian population, REF1 and REF2 to the two reference populations and GEN to the generalised movement model (see “Methods”). The parameters *k* (dimensionless), *D* (m^2^ day^−1^), *m* (day^−1^ ), and *p* (dimensionless) refer to relative habitat preference, movement rate, mortality and capture probability, respectively. The Subscripts BH, HQM, LQM and R stand for breeding habitat, high-quality matrix, low-quality matrix and the river, respectively (see “Methods”).
